# Micro-computed tomographic evaluation of dentinal defects after root canal preparation with hyflex edm and vortex blue rotary systems

**DOI:** 10.4317/jced.54853

**Published:** 2018-09-01

**Authors:** Jyothi Mandava, Rajiv-Kumar Yelisela, Sampath-Kumar Arikatla, Ravi-Chandra Ravi

**Affiliations:** 1 Professor & Head of the department, MDS in Conservative Dentistry and Endodontics). Gitam Dental College and Hospital; Visakapatnam; 2 Senior lecturer, MDS in Conservative Dentistry and Endodontics). Gitam Dental College and Hospital; Visakapatnam; 3 Senior lecturer, MDS in Conservative Dentistry and Endodontics). Gitam Dental College and Hospital; Visakapatnam; 4 Associate Professor, MDS in Conservative Dentistry and Endodontics). Gitam Dental College and Hospital; Visakapatnam

## Abstract

**Background:**

To evaluate the incidence of dentinal microcracks formation after root canal shaping procedures with HyFlex EDM and Vortex Blue rotary systems comparing with that of hand instrumentation using micro-computed tomography.

**Material and Methods:**

Mandibular first molar teeth (n=60) having 50 to 200mesial root curvature were scanned using high resolution micro-CT imaging before root canal preparation to identify the presence of dentinal defects. Post-instrumentation cross-sectional images were taken and increased number and type of root defects were assessed and recorded. The data was statistically analysed by using chi-square test and McNemar’s test at a significance level of 5%.

**Results:**

HyFlex EDM has shown greater increase in post instrumentation dentinal defects. Both rotary systems exhibited increased dentinal defects formation at coronal, middle regions which is statistically significant (*P*=0.042) when compared to apical region.

**Conclusions:**

HyFlex EDM has shown greater increase in post instrumentation dentinal defects followed by Vortex Blue rotary system and hand instrumentation resulted minimal defects.

** Key words:**Craze lines, dentinal microcracks, micro–computed tomography, nickel-titanium files, root canal preparation.

## Introduction

Successful endodontic treatment requires predictably shaped root canals to facilitate three dimensional obturation. Nickel-titanium (Ni-Ti) instruments shape the root canals efficiently and reduce the incidence of procedural errors as they have 2 to 3 times more flexibility when compared to stainless steel instruments (1). Inorder to reduce operator fatigue, treatment time and to create a continuously tapered conical flare preparations, several Ni-Ti rotary systems have been evolved during the last decade. Root canal shaping procedures and rotary instrumentation have been claimed as potential causes to induce formation of root dentinal cracks which can be extended as vertical root fracture(VRF) under functional load (2,3). Large tapered rotary instruments removes substantial amount of dentin especially in teeth with curved roots or oval canals. Lateral forces developed during mechanical root canal preparation can induce strain on the canal walls and may cause microcrack formation or fracture propagation (4). Mechanical stress developed with rotary instrumentation is strongly dependent on canal curvature and also influenced by flexibility, diameter and rotary kinematics of the endodontic instruments.Recently developed heat treated instruments are more flexible, resistant to cyclic fatigue and has shown to develop fewer dentinal defects compared with traditional NiTi rotary instruments (5,6).

The Hyflex EDM file (HEDM; Coltene / Whaledent AG, Switzerland) using electrical discharge machining (EDM) manufacturing process create a rough and hard surface of the file, resulting in superior fracture resistance and improved cutting efficiency (7). HEDM file has a tip size of 25 with a constant 0.08 taper in the apical 4mm of the instrument but reduces progressively upto 0.04 taper in the coronal portion. It has 3 different cross-sectional zones with a triangular cross-section at top, trapezoidal in middle part and rectangular in the apical part of the instrument working portion (8). It was reported that, this variable cross-sectional design contributes to lesser dentinal cracks formation (5).

The Vortex Blue rotary system (DenTsply Tulsa Dental speciality, USA) uses a new proprietary method during NiTi wire processing and produces a distinctive “blue colour” titanium oxide surface layer. These instruments produced by complex heating and cooling procedures, exhibit greater fatigue resistance and improved flexibility with controlled shape memory (9). They have variable helical angle cutting blade with triangular cross-section. The lower helical angle (less flutes) in the coronal portion of the file facilitates efficient debris removal and the higher helical angle (more flutes) in the apical portion facilitates increased strength with minimal chance for dentinal cracks formation (5).

Most of the previous studies reported that instrumentation with hand files produced no or minimal dentinal defects (2,10,11), but one recent study claimed that manual instrumentation caused more dentinal defects than protaper rotary system (12). Because of these inconsistent reports about their biomechanical impact on root dentin, the objective of the study was to evaluate and compare the incidence of dentinal microcracks formation in moderately curved root canals, after performing shaping procedures with HyFlex EDM or Vortex Blue systems comparing with that of hand NiTi instrumentation, using micro-computed tomography. The Null hypothesis was there will be no difference in dentinal defects formation among the rotary systems tested.

## Material and Methods

-Sample selection

One hundred non-carious, mandibular first molar teeth were radiographed in both directions to ensure that all the mesial roots had two separate canals. Teeth having root canal systems with reduced pulp spaces, pulp stones or calcified canals were discarded. All roots were inspected under 12X magnification to ensure that they did not have root caries, hypercementosis, open apices, cracks or craze lines. Collected teeth were recently extracted and their use in research was approved by the local biomedical research ethics committee (D158601033). Only teeth in which radii of curvature ranged between 2.5mm to 6.3mm and the angle of root canal curvature between 50 to 200 (moderately curved) were selected. As a result, only 60 specimens with moderate mesial root curvatures were selected and stored in 0.1% thymol solution at 50C. Sample size was estimated using G* Power 3.1 software (Heinrich Heine, Dusseldorf, Germany). Twelve samples were indicated as the minimum ideal size required for observing the effect of instruments on root dentin with an alpha error probability of 0.05.

For all the 60 selected teeth, coronal part of the tooth was removed 1mm above the cemento-enamel junction, using a flexible diamond disk. The mesial roots were resected by placing a vertical cut at the furcation area and the distal roots were discarded. Baseline images were taken with a micro-CT scanner (X-radia Versa 500, Ziess, Germany), to detect the presence of pre-operative microcracks or craze lines in the root dentin. The long axis of the roots were adjusted so that they are perpendicular to the X-ray beam to provide scans in the same sagittal plane. The scanning was performed according to the following parameters: a rotation of 3600 in vertical axis with a rotational step of 0.50, and the camera exposure time of 500 milliseconds. The specimens were imaged at an isotropic resolution of 30μm, and filtered with 1mm thick aluminium filter. Three dimensional images were generated and reconstructed using the software (Scout and ScanTM control system 10.7.3679.13921 Ziess, Germany).

The pre-instrumentation (baseline) images of all root samples were analysed, and the cross-sectional area of each specimen in which a dentin defect had been observed were recorded using the following parameters;

1. The location of the crack observed on the root (ie, cervical, middle, or apical thirds of the root).

2. Whether the defect observed in the root dentin was complete or incomplete crack or craze line.

-Root canal preparation 

The root samples were wrapped with aluminium foil of thickness 0.2mm, and embedded in metal matrices containing autopolymerizing acrylic resin (RR cold cure powder/liquid, DPI, Mumbai, India). The roots were removed from the matrices, the foil was detached and an polyether material (Ad-Sil, Prime dental products pvt Ltd. Mumbai) was manipulated and inserted into this space to simulate periodontal ligament. The roots were repositioned in acrylic resin blocks and were randomly divided into three groups (n=20 each), according to the NiTi file system used for shaping the canals.

Group 1: (n=20) Hand Ni-Ti flex files (DenTsply Maillefer, Ballaigues, Switzerland) 

The coronal part of the canals were flared using #2 gates-glidden drills (DenTsply Maillefer). The working length was estimated using #10 k file and the canals were enlarged upto #30 master apical file in a crown-down manner in both mesio-buccal and mesio-lingual canals.

Group 2: (n=20) HyFlex EDM rotary file system with variable taper

Canal patency was verified in both the canals. Orifices were widened with HyFlex EDM orifice opener #25/.12 with500rpm rotational speed at 2.5 N-cm torque using the Endomate DT (NSK, Nakanishi Inc, Tochigi, Japan) endomotor. Then the canals were prepared sequentially with #10/.05glide path file followed by Hyflex CM #20/.04 and #25 Hyflex shaping one file having variable taper,according to the manufacturers recommendations upto the working length.

Group 3: (n=20) Vortex Blue rotary file system

After establishing initial glide path, the working length was estimated in both the root canals. Vortex Blue files were usedin a sequential order with # 15/.04,# 20/.04 and # 25/.04 files at 500rpm at a torque of 1.0 N-cm and worked upto the working length.

Between each instrumentation, recapitulation was done using #10/.02 hand files to maintain the glide path. During instrumentation, the canals were irrigated with 5ml of 3% sodium hypochlorite using 27gauge needle (NaviTip, Ultradent, South Jorden,USA). After completion of instrumentation, each root canal was irrigated with 17% ethylene diamine tetra acetic acid for 1 minute to remove the smear layer. Final flush was done with 2 ml of normal saline. Each hand or rotary file was used to prepare only 4 canals and all the shaping procedures were performed by a single operator. Throughout the experimental period, all roots were kept moist in distilled water to avoid any artifact by dehydration.

-Dentinal microcrack evaluation

**Figure 1:**
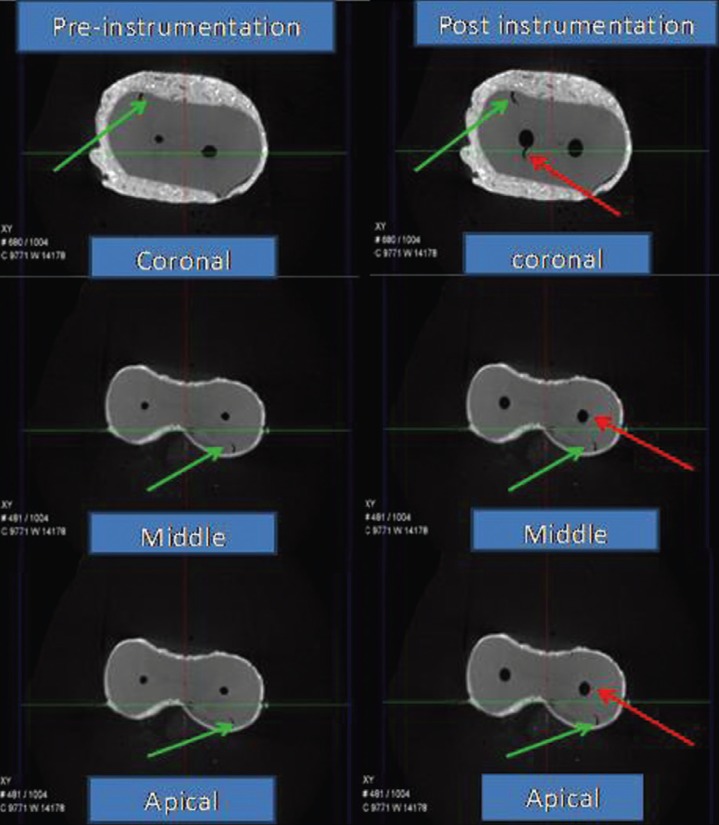
Micro-CT images Ni-Ti Flex file group (Green arrow showing pre-instrumentation defects and red arrow post instrumentation defects).

**Figure 2:**
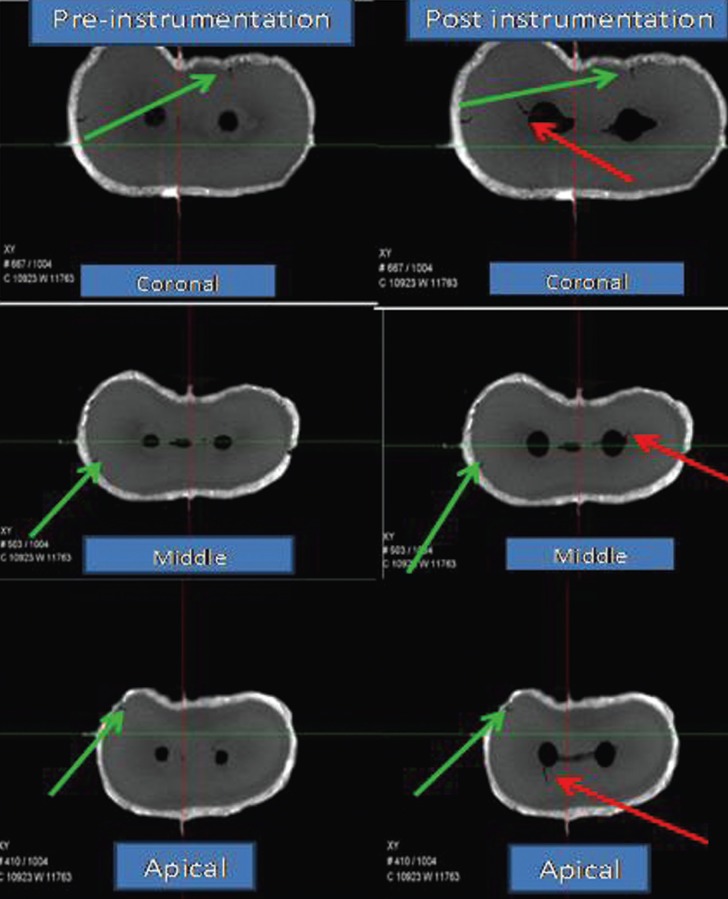
Micro-CT images of dentinal defects in HyFlex EDM rotary system group.

**Figure 3:**
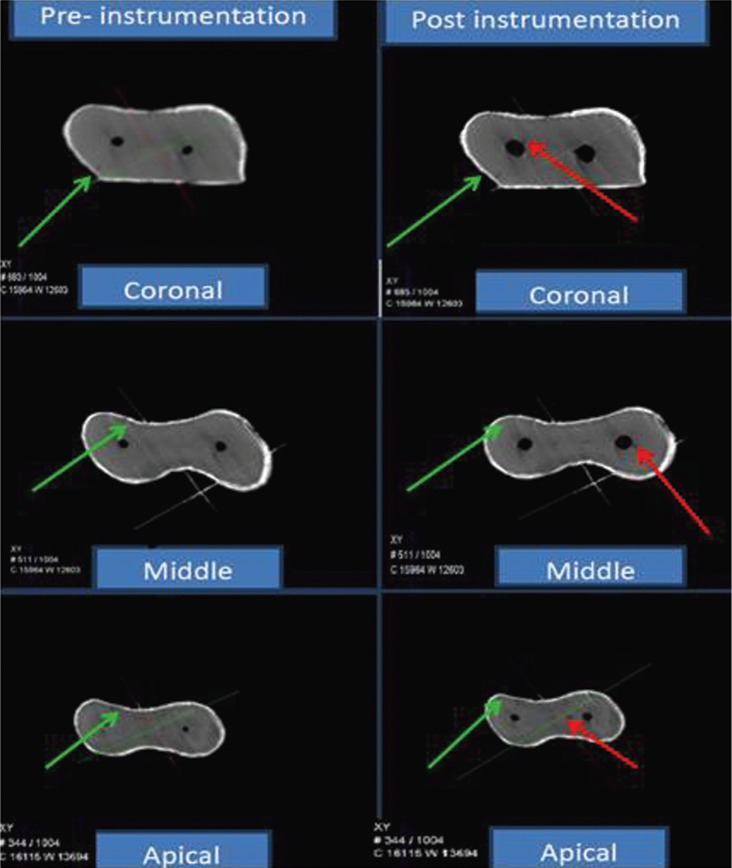
Micro-cracks formation with Vortex Blue rotary files.

After chemo-mechanical preparation of the root canals, all root specimens were imaged again through micro-CT scanner. The reconstructed 3-dimensional images of the samples before and after preparation were compared twice by 2 blinded observers to identify the presence of dentinal defects (Figs. 1-3).

The identification of a defect was based on the following definition:

a) Incomplete crack- if the crack line is extending from the canal wall into the dentin without extending on to the external surface of the root.

b) Complete crack- if the crack line is extending from the root canal wall to the outer surface of the root.

c) Craze lines are the lines observed in the root dentin that didn’t reach any surface of the root or extend from the outer surface into the dentin but didn’t reach the canal wall (16).

The number of microcracks observed were determined as a percentage for each group. The data of defects observed were subjected to statistical analysis using SPSS/PC software program (Version 20; IBM; Chicago; IL).A bivariate analysis was performed by using chi-square test and McNemar test to determine before and after instrumentation differences. Level of significance was set at ≤ 0.05.

## Results

The percentage of pre-instrumentation dentinal defects observed in all the groups were ranged between 15% to 22% ( Table 1). Both the rotary systems exhibited statistically significant increase in the percentage of dentinal defects after instrumentation (*P*=<0.05), whereas minimal increase in defects were observed with hand Ni-Ti Flex files (*P*=0.999) which was not statistically significant. No significant difference was seen between the two rotary systems (*P*=0.3060) ( Table 2). Among the two rotary systems, Hyflex EDM has shown greater increase in post instrumentation dentinal defects.

**Table 1:**
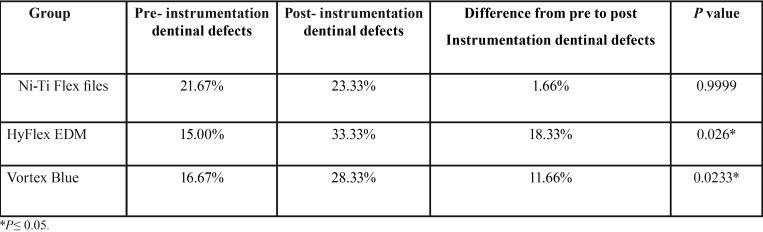
Comparision of pre and post instrumentation percentage of defects in root dentin among the groups.

**Table 2:**
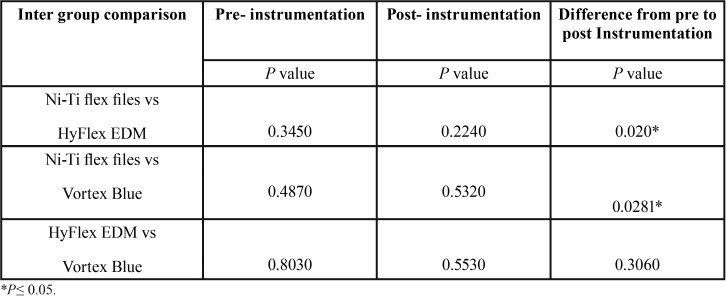
Inter group comparison ofP values obtained by Mcnemar test.

Incidence of incomplete microcracks formation was significantly high for Hyfex EDM (*P*= 0.0232) and vortex blue (*P*=0.0447) systems when compared to NiTi flex files ( Table 3, Table 4). No significant difference was found between the groups in craze lines and complete cracks formation.

**Table 3:**
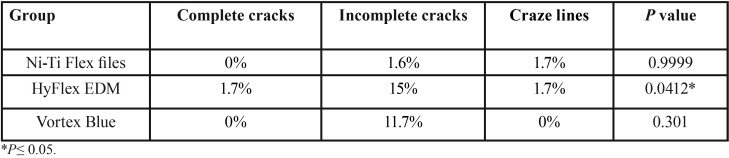
Comparison of type of dentinal defects increased percentage (%) from pre to post instrumentation.

**Table 4:**
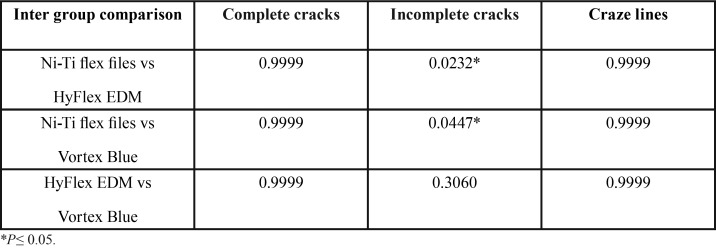
Inter group comparison of type of dentinal defects P values obtained by Mcnemar test.

Hyflex EDM has exhibited statistically significant increase in dentinal defects at coronal, middle region (*P*=0.042) ( Table 5, Table 6), when compared to apical region. Vortex Blue system also increased the incidence of dentinal defects more in coronal and mid root regions, but the difference was not statistically significant (*p*=0.094).Hand instrumentation has shown minimal increase in the percentage of post instrumentation defects at coronal region only, which was not significant (*p*=0.999) statistically.

**Table 5:**
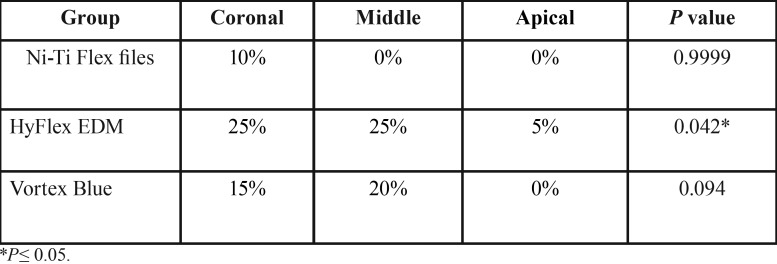
Comparison of Pre to Post instrumentation increased percentage of defects at different root regions.

**Table 6:**
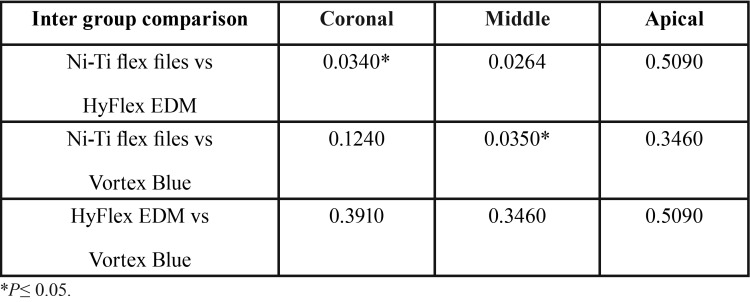
Inter group comparisionof P values obtained by Mcnemar test .

HyFlex EDM rotary files exhibited statistically significant difference in the formationof dentinal defects at coronal and mid-root regions (*P*=0.034 & 0.0264 respectively) compared to Ni-Ti hand file instrumentation. The number of dentinal defects formed with the Vortex Blue system were significantly high only in mid-root region (*P*=0.0350) compared to Ni-Ti Flex file system. Therewas no difference in the defects formation between the two rotary systems at any root region.

## Discussion

Over the past two decades rotary NiTi based preparations became the main stream approach to enlarge the root canal space mechanically. Studies reported that, there is a casual relationship between rotary instrumentation on dentinal microcrack formation (10,11). The first authors to correlate the dentinal microcracks formation to root canal preparation by motor driven NiTi instruments were Bier et al and Shemesh et al. (2,3).They attributed this to instrument design concepts and kinematics.

Commonly used in-vitro method to evaluate the post-instrumentation microcracks formation is by assessing the pictures taken under magnification after sectioning of the root (13,14). Limitations of the sectioning method is the possibility of having false positive results, as extraction and sectioning procedures might create or propagate existing dentinal defects. Micro-computed tomography method could be applied both quantitatively and qualitatively for three dimensional assessment of root canal system. Micro-CT enables the visualization of the pre-existing defects and their precise location throughout the root and this improves the validity of the experiment because each specimen acts as its own control (15,16).

Moderately curved roots were selected in the study, as it was demonstrated that shaping the root canals with a smaller radius and greater curvature increased the incidence of dentinal microcracks formation (10). Due to constricted anatomical configuration of mandibular molar mesial canals, more stresses may develop on the dentinal surface during mechanical preparation, increasing the potential to develop cracks (15).

Micro-CT image analysis was done for examining the hundreds of cross-sectional pre and post instrumentation images for identifying the dentinal defects. This method facilitates the defect localization while avoiding damage to the tooth structure, to obtain reliable results (15,17,18).

Pre-instrumentation micro-CT images revealed the presence of dentinal defects ranging between 15 % to 22%. This finding is in agreement with previous micro-CT studies (11,15), where they found root dentinal defects ranging between 16.7% to 34.6% before root canal preparation. According to Pradeep *et al.*, pre-existing root dentinal defects in mandibular teeth were significantly high (10.3%) than in maxillary teeth (2.9%) and were typically found in the cervical and middle regions of root surface in the mesio-distal direction. It was assumed that these defects in unprepared teeth were either as a result of forces induced during extraction or due to excessive occlusal functional loads during life before extraction or due to aging process (19).

Hand Ni-Ti instrumentation caused minimal increase (1.66%) in dentinal defects formation at coronal level. Use of gates glidden drills for coronal root canal flaring might have resulted in the formation of these defects. Similar findings were observed in the previous studies (20,21) where the samples prepared with gates glidden drills exhibited microcracks. To complete a preparation with rotary Ni-Ti files significantly more rotations are required as compared to hand files contributing to the formation of more dentinal defects. In accordance with the observations of the previous studies (11,14) both the rotary systems demonstrated significant increase in the percentage of dentinal defects after canal instrumentation. Contrary to this finding Bayram *et al.* (5) claimed that the instrumentation with Vortex Blue and HyFlex EDM rotary files developed no new microcracks and they attributed this finding to the heat treated structure of these instruments, which gives extra flexibility to the files. Straight root canals were used in their study, whereas moderately curved mesial roots (50 to 200) used in the present study might explain the possible reason for increase in dentinal microcracks formation. These findings differ from the observations by De-Dues *et al.* studies (15,17,18), where they found lack of correlation between dentinal microcracks formation and root canal preparations with rotary systems.

Among the two rotary systems, HyFlex EDM has shown greater increase in post- instrumentation dentinal defects. Formation of defects might be associated with the tip design, cross-sectional geometry, taper type (constant or progressive), flute form and pitch (constant or variable) of the rotary file (22).

HyFlex EDM file has 3 different (rectangular in the apical part and two different trapezoidal cross-sections in the middle and coronal part) cross-sectional zones over the entire working length of the instrument. This design feature might generate screwing effect and dangerous taper lock can maximize the contact between the file and the dentin causing formation of dentinal defects.

In the present study most of the cracks were located at the cervical and midroot regions. The reason for this finding might be explained by previous *in vivo* strain gauge experiment results (23), that reported the occurrence of maximum functional strain distribution at the coronal and mid- root surfaces.

In the present study both the Ni-Ti rotary systems exhibited higher percentage of incomplete cracks. Similar findings were observed in Burklein S *et al.*(13) study where they found incomplete cracks in the range of 15% to 25%. The current study results are correlating with the findings of Arias *et al.* (24), in that the microcracks formed after root canal instrumentation were in the mesio-distal directon. Based on the investigations on VRF in endodontically treated teeth, Sugaya *et al.* (25) reported that most apical fractures may be in mesiodistal or buccolingual direction and generally VRF have been reported to be in buccolingual direction.

It was reported that crack propagation was continuous in root slices even after 1 month of storage with no further stress over dentin (26) and thus the baseline status of the sample is crucial for reliability. According to American association of tissue bank recommendation, a storage temperature of extracted teeth at -200 C was not maintained in this study. Likewise, using a human cadaver maxilla, a study found no difference in the number of microcracks before and after root canal rotary instrumentation (17). Therefore, future investigations using human cadaver models with a noninvasive micro-CT imaging methods in curves root canals, will provide a more profound information regarding the formation of dentinal microcracks.

## Conclusions

Within the limitations of this invitro study, root canal preparations with HyFlex EDM and Vortex Blue rotary systems were found to induce the formation of new dentinal microcracks in the moderately curved mesial root canals of mandibular molars. Endodontists should be aware of the safety of newly introduced rotary systems in regard to the creation of dentinal defects.
